# Time-frequency domain analysis of investor fear and expectations in stock markets of BRIC economies

**DOI:** 10.1016/j.heliyon.2021.e08211

**Published:** 2021-10-19

**Authors:** Peterson Owusu Junior, Anokye M. Adam, Emmanuel Asafo-Adjei, Ebenezer Boateng, Zulaiha Hamidu, Eric Awotwe

**Affiliations:** a Department of Finance, School of Business, University of Cape Coast, Cape Coast, Ghana; b Department of Marketing and Supply Chain, School of Business, University of Cape Coast, Cape Coast, Ghana; c Directorate of Finance, University of Cape Coast, Cape Coast, Ghana

**Keywords:** Financial markets, Interdependence, Asymmetric volatility, Financial instability, COVID-19 pandemic, Comovements

## Abstract

The purpose of this study is to provide insight into the lead-lag relationships between the BRIC stock index and its constituents. In addition, we assess the comovements between the US volatility index (VIX) as a measure of investor uncertainty and fear and stock returns of BRIC economies. Therefore, the bi-wavelet and wavelet multiple correlations approaches are utilised. Findings from the bi-wavelet technique indicate that there are high interdependencies between the BRIC index and its constituents throughout the time-frequency domain. In addition, comovements between the BRIC index and its constituents was positive and significant. Notwithstanding, we find the BRIC index to be the first variable to respond to shocks when all the study variables were considered in the wavelet multiple cross-correlations. Similarly, the stock market of Brazil is the next to respond to shocks. On the other hand, the stock market of Russia lags in the long-term when the BRIC index was excluded from the wavelet multiple cross-correlations. We also find a uni-directional causality between the VIX and the BRIC stocks in the medium-, and long-terms. Specifically, the US VIX significantly drives the BRIC stocks and considered to be negative. Findings from the study imply that global investors can select any of the stock markets in BRIC to allocate their investments due to their strong interdependencies which may facilitate trade and investments. However, portfolio diversification, safe haven or hedge benefits within this region may be minimal due to their high integration with the BRIC index which demonstrates positive significant comovements. The findings present relevant inferences for portfolio diversification, policy decisions, and risk management schemes. It is recommended that investors hedge against volatilities in the BRIC stock markets using the US VIX.

## 1 Introduction

The stock markets of BRIC have gained massive interest from researchers, policymakers and global investors due to the markets’ usefulness for portfolio diversification, safe haven and hedge, aside from being active and vibrant (Swamy and Narayanamurthy, 2018; Sharma et al., 2019; Rehman, 2021; Shahzad et al., 2021). For this reason, BRIC markets have been touted to be one of the drivers of global growth (Sachs, 2003; Grima and Caruana, 2017; Rehman, 2021; Mensi et al., 2014; Kalu et al., 2020). In the light of increasing foreign investments as well as the free flow of capital, BRIC economies are most likely to maximise their potential for development. BRIC member nations have increased their market liquidity and depth, boosted investor protection, and improved their regulatory standards as a result of more emerging market economies (EMEs) are undergoing financial liberalisation and economic integration (Mensi et al., 2017; Sharma et al., 2019).

The financial market integration theory has, therefore, made possible the financial markets in an economy to become more closely related with those in other economies around the world (Asafo-Adjei et al., 2021). Financial integration, to begin with, is the consequence of institutional, economic, and political reforms. Global investors' ability to acquire domestic assets, as well as local investors' ability to access international investment opportunities, both legitimately and illegally, is critical to integration (Arouri and Foulquier 2012; Shadlen, 2005). Access to global international investment options, whether by legal means, home-grown diversification, or illegal means, enhances the exposure of domestic financial assets to the global, and so raises the level of national stock market integration (Arouri and Foulquier 2012). Investors’ risk preference, relative optimism, and information perception are all behavioural characteristics that might influence the willingness to invest overseas (Arouri and Foulquier, 2012). This is not far from the behaviour of BRIC economies. BRIC economies have become increasingly important to the global investment community in recent years. This is true for a variety of reasons, including expectations of growing dominance in the international arena, as well as significant shifts in capital flows into their markets (Sensoy et al., 2014; Swamy and Narayanamurthy 2018), despite the capital flight due to tapering by the US Federal Reserve. Their markets also share a conception of development that is more partnership-orientated than donor-recipient focused in process (Piper, 2015). The BRIC countries have gained much attention from domestic and global investors, portfolio managers and policymakers due to the improvement of their size and volume of trade.

BRIC economies are among the top emerging market investments for the US and international investors seeking ideal portfolio allocation and international diversity (Bhuyan et al., 2016). Assets from the BRIC markets can produce good returns during times of stress and may provide a safe haven for returns during times of uncertainty, especially, the COVID-19 pandemic (Shahzad et al., 2021). Global investors seeking to diversify and generate higher returns on investment possibilities offered by BRIC economies must know the characteristics of these individual markets as well as how they interact with one another. Despite this, the BRIC economies are not homogeneous and are at various levels of growth, with some being more developed than others at different times and with varied frequencies. In the same way, their respective financial markets may have distinct levels of depth, regulatory systems, and market microstructure patterns. According to a study by Kishor and Singh (2017), BRIC stock market movements are not uniform and are dependent on specific markets. Accordingly, the contribution of each constituent of BRIC markets to the BRIC index may not be identical.

The BRIC index is a market capitalisation-weighted index that tracks the performance of equity markets in the four emerging nations of Brazil, Russia, India, and China (MSCI, 2021). The index, which has about 916 constituents, covers about 85% of each country's free float-adjusted market capitalisation (MSCI, 2021). This index has experienced rapid fluctuations over time, especially, during market stress periods such as the aftermath of the 2007–2009 Global financial crisis, the 2009–2012 Eurozone crisis, US-China Trade tension, the COVID-19 pandemic, etc.

BRIC countries, like other emerging markets, are vulnerable to macroeconomic and global market conditions (Chittedi, 2010; Grima and Caruana, 2017; Bouri et al., 2018a, Bouri et al., 2018b). While internal variables play an important part in driving economic and financial circumstances in BRIC economies, there is a lot of indication that external influences are driving several of these countries' economic conditions (Bouri et al., 2018a, Bouri et al., 2018b). For example, BRIC countries were affected by the global financial crisis (GFC) and had erratic capital flows and stock market performance as a result. Several studies show that BRIC economies are becoming more integrated with developed economies, with evidence of large financial flows from the latter to the former (Lehkonen and Heimonen, 2014; Bouri et al., 2018). As a result, the BRIC markets' vulnerability to changes in global, economic, and financial conditions has been investigated (Lehkonen and Heimonen, 2014; Grima and Caruana, 2017; Bouri et al., 2018a, Bouri et al., 2018b; Bouri et al., 2018). The impact of US macroeconomic conditions, notably changes in US stock indices, on the stock markets of important emerging countries, such as BRIC, has been well documented in both tranquil and turbulent eras (Singh and Singh, 2018; Karanasos et al., 2021). Intuitively, worsening macroeconomic conditions hurt stock market performance in BRIC nations by reducing exports and capital inflows (Bouri et al., 2018). As a result, global shocks, particularly those initiated in the US stock market, can be conveyed to the stock markets of the BRIC economies.

From a theoretical standpoint, Minsky (1975, 1986, 1992) proposed the financial instability theory, which linked financial market fragility to endogenous speculative investment bubbles. This hypothesis claims that economic tranquillity and stability are not self-sustaining. Accordingly, stability could lead to more optimism which eventually leads to more borrowing in stocks and assets. Over time, there is a transformation from a stable financial system to a fragile system. Thus, consequently, stable and booming markets drive blindness for increasing risks (Thielen, 2016). In addition, arguments on asymmetric volatility in the discussion of the US VIX and stock markets cannot go unnoticed. Fluctuations in market volatilities influence investors’ portfolio choices either by altering the trade-off between risk and return or their predictions of future market performance. According to Chen (2018), investors desire to hedge against market volatility since rising volatility does not incentivize investment opportunities. Suffice to say, phases of high volatility tend to concur with drawdowns in stock markets which may reduce investors' confidence (Campbell and Hentschel, 1992).

Rapach et al. (2013) advocate that the US equity returns are a significant predictor of equity returns in both developed and developing countries. According to Sarwar (2012), there is an inverse relationship between stock market returns in large emerging countries like the BRICS and market uncertainty in the US, as measured by the volatility index (VIX). In addition, Sarwar and Khan (2017) discovered that rising US market uncertainty reduces emerging market returns while increasing return variance. Furthermore, except for Russia, Kishor and Singh (2017) discovered that news of a US stock market drop had a significant impact on BRIC stock markets. This shows that uncertainty in the US may have an impact on the stock markets of the BRIC countries. The majority of previous studies have focused on the interconnectedness between the BRIC stock markets (Chittedi, 2010; Dasgupta, 2014; Heliodoro et al., 2020), whilst others have concentrated on the return and volatility of the US and BRIC markets (Rapach et al., 2013; Bhuyan et al., 2016; Sarwar and Khan, 2017). However, a study that analyses integration between the BRIC index and its constituents while gauging investor expectations and fear has received scant attention. But prior studies that assess information flow between stock market index and its components have confirmed the importance of addressing this issue in various stock markets and regional blocs (Kwon and Yang, 2008; Osei and Adam, 2020). In addition, the lead-lag correlations across the implied VIX of US and BRIC markets, including the COVID-19 pandemic era, while differentiating between short-, medium-, and long-run frequency domains, have received little or no attention. This is necessary because, the COVID-19 pandemic has disrupted many financial and economic activities. In this case, a study that reveals both the time and frequency discussion on stock markets is worthwhile (Sharma et al., 2021; Kamaludin et al., 2021). Furthermore, the examination of volatilities enables investors to adjust their risk preferences (Prasad et al., 2020), and it is hoped that regional analysis can help investors better understand the regional composition of volatility in their portfolios.

Accordingly, studies conducted on the VIX of the US and BRIC stock markets assess their co-movements with less concentration on the time and frequency domains. The intrinsic complexity in time series has increased the time-frequency domain analysis, thus, wavelet analysis is becoming a usual instrument for examining localised variations of power within a time series to define both prevailing modes of variability and how the modes change in time through decomposition (Owusu Junior et al., 2018; Tweneboah et al., 2019; Frimpong et al., 2021; Asafo-Adjei et al., 2021; Diallo et al., 2021). The application of bi-wavelet, wavelet multiple correlation (WMC) and wavelet multiple cross-correlations (WMCC) are, therefore, important in this study. While bi-wavelet coherence is a technique that shows the correlation between two variables, the resulting wavelet transformation coherence of several variables is ideal for WMC and WMCC (Kahraman and Ünal, 2016; Owusu Junior, Adam and Tweneboah, 2017). Specifically, we seek to examine the lead-lag relationships at the diverse times and frequencies pictorially from 2012/12/11 to 2021/05/28 using the bi-wavelet analysis.

We further assess the degree of integration among the VIX of US, BRIC constituents and the BRIC index concurrently by applying the WMC and WMCC. This is necessary to determine the extent of volatility spillover on the BRIC markets based on the BRIC markets' degree of integration. Again, market participants react irrationally to information at different times, resulting in very noisy market data. To correctly define this issue, the presence of different frequencies proffer stock market participants’ diverse investment time scales. This is in line with the heterogeneous market hypothesis (HMH) as indicated by Müller et al. (1997). Also, the adaptive market hypothesis (AMH) engineered by Lo (2004) suggests that markets evolve – due to events and structural changes, adapt – and market efficiency varies in degree at different times. The AMH has been revealed by Lekhal and El Oubani (2020) to be essential within financial markets of emerging economies, and thus, assessed to be time-varying. Therefore, time-frequency domain analysis contributes to the reduction of weak signals to maintain the true signals (Owusu Junior, Adam and Tweneboah, 2020; Asafo-Adjei et al., 2021).

The study contributes to literature in many ways. First, we investigate the comovements between the constituents of the BRIC stock markets and the BRIC index via the bi-wavelet technique. This would determine the contribution of each constituent towards the development of the BRIC index in both time and frequency to reveal the extent of heterogeneity among them. Second, the reverse causality from the BRIC index to the constituents is also considered since it is established that the development of a market index corresponds to a significant flow of information to its constituents (Kwon and Yang, 2008; Osei and Adam, 2020). Third, we assess the lead-lag relationships between the US VIX and BRIC stock markets (both constituents and the index) in time-frequency, via the bi-wavelet approach. It is expected that the directional comovements between the VIX as a measure of investor expectation and fear and each of the BRIC markets is negative, as external policy uncertainty could play a further role leading to drawdowns of financial markets (Adam, 2020; Asafo-Adjei et al., 2020). In addition, the intensity of negative comovements between the VIX and the BRIC markets may be substantial during the COVID-19 pandemic which corroborates the tenets of the efficient market hypothesis. This is because, market prices of most stocks are correctly priced based on the information available at that time (Owusu Junior et al., 2021). If indeed, there is efficiency in the markets, the negative comovements would not follow a specific pattern. However, if the BRIC stock markets are highly integrated as indicated by extant literature (Chittedi, 2010; Dasgupta, 2014; Heliodoro et al., 2020) to enjoy their long-standing relationships, we expect limited volatility transmission within the combined stock market returns of BRIC. Thus, there may be less expectation for the volatility index to drive the highly integrated stock markets. This is, therefore, captured by the WMC and WMCC which address the degree of interdependencies among all the variables simultaneously. As indicated by Tan et al. (2019), time scale analysis in emerging markets cannot be ignored due to their increasing level of trade and investments. In this regard, the current study is among the very few empirical studies that assess the comovements between and/or among the stock markets of BRIC constituents, BRIC index, and the US VIX via the wavelet approach, and inclusive of the COVID-19 pandemic.

The bi-wavelet technique revealed that there is high interconnectedness between the BRIC index and each of its constituents, and were concentrated in the short-, medium-, and long-term. In addition, comovements between the BRIC index and its constituents were positive and significant throughout the wavelet scales and time. Thus, in times of uncertainties, drawdowns in the performance of the constituents are, however, reflected in the BRIC index. It goes to suggest that the BRIC index is a true representation of its constituents. Notwithstanding, we found the BRIC index to be the first variable to respond to shocks when all the study variables were considered in the WMCC. Further, we found investor expectation and fear to have an adverse relationship with BRIC economies in the medium-, and long-term, especially, beyond 2019. This may be attributed to the COVID-19 pandemic which has distorted global financial and economic activities. As a result, BRIC returns are typically connected with higher (downward) adjustments of the US volatility index. Contrarily, the US VIX could not drive the stock markets of BRIC when the wavelet multiple analysis was considered. This is not overwhelming because, when there are strong interdependencies between BRIC markets, participants may benefit from their long-term relationships.

The next section of the study presents a brief review of existing empirical literature. This is followed by the research methodology, and presentation and discussion of results. Finally, the study details out the conclusion and provides some implications for policy and practice.

## 2 Literature review

Recently, the comovements between stock markets in BRIC economies and stock returns of developed markets have received enormous attention among investors and academics due to the implications for portfolio construction and optimization. Consequently, some studies have shed light on the degree of integration between these stocks. However, the existing empirical evidence has been ambiguous.

Lehkonen and Heimonen (2014) examined time-scale dependent comovements between BRIC stocks and stock markets of developed economies. By employing a wavelet decomposition and a dynamic conditional correlation framework, their findings indicate that BRICS provide portfolio diversification benefits to stock markets in the developed economies including the US. Further, Bhuyan et al. (2016) examined the information and dynamic spollovers between the US stock market and BRIC stocks. Using the GARCH model, the empirical evidence revealed that the US stock market exhibit significant volatility and mean return spillover effects on the BRIC stocks while the Chinese stock market also has a mean spillover effect on both the US and Indian stock markets.

Sarwar (2012) documents an inverse relationship between stock returns of emerging markets and the VIX. Similarly, Sarwar and Khan (2017) also examined how stock market returns in Latin America and the broader sense, emerging markets respond to the VIX prior, during, and after the GFC in 2008. Their empirical evidence revealed that the VIX exhibited a direct linkage with return volatility in these markets but affected stock returns of the markets negatively. Furthermore, Dutta (2018) employed the ARDL bound test and the Toda-Yamamoto Granger causality test to examine the relationships across implied volatilities in the US, Brazil, and China. He revealed a bidirectional causality between the VIX and implied volatility of the Chinese stock market. Nevertheless, the study found no predictive power of the implied volatility of the Brazilian stock markets to the VIX.

Thus, the consensus in the academic literature is that the US stock market has become a significant predictor in assessing stock returns, particularly in emerging BRIC economies. However, the nature of the comovements between the US stock market and the BRIC stocks have been unclear in the literature as the extant evidence divulge contrasting findings. Moreover, clarity in the extent of integration and connectedness of the financial markets is very crucial to facilitate trade and investments, especially during the pandemic. One possible reason that may account for the disparities in outcomes of the existing studies is that these studies fail to take into consideration time and frequency domain in tandem in their analysis. In addition to this, little or no attention is given to the BRIC index in assessing the finance and economic integration with its constituents, and the contribution of each constituent to the index. This is imperative to reveal the extent of similarities among the constituents to facilitate policy and investment decisions.

## 3 Methodology

We present the wavelet techniques (bi-wavelet and wavelet multiple) are essentially employed in this study to assess the time-, and/or frequency comovements of the study variables, and also determine their lead/lag relationships.

### 3.1 Bi-wavelet

#### 3.1.1 Continuous wavelet transform (CWT)

The fundamentals of wavelet analysis comprise two factors: time or location (i) and scale (s), expressed in Eq. (1)

(1) $${\text{ψ}}_{ί,s}\left(t\right)=\;{\sqrt{s}}^{-1}\;\text{ψ}\;\left(t-i\right)\left(s^{-1}\right),\;\text{ψ}\left(\cdot \right)\in L^{2}\;\left(\text{R}\right)$$

where ${\sqrt{s}}^{-1}$ is the normalization factor, guaranteeing that the unit variance of the wavelet $||{\text{ψ}}_{i,s}\left(t\right)|{|}^{2}=\;1;i\;$ || is the location factor, offering the precise place of the wavelet; and s is the scale dilation factor, describing the stretched nature of the wavelet. The Morlet wavelet can be precise in Eq. (2)

(2) $$\varphi^{M}\;\left(t\right)=\;\pi^{-1/4}e^{i\omega_{o}t}e^{-t^{2}/2}$$

where the dominant frequency of the wavelet is denoted by $\omega_{o}$. We set $\omega_{o}$ at 6 Rua and Nunes (2009).

A time series *x (t)* based on a selected mother wavelet can be decomposed (Li et al., 2020) as

(3) $$w_{x}\left(i,s\right)=\;\overset{\infty }{\underset{-\infty }{\int }}x\left(t\right)\;{\sqrt{s}}^{-1}\text{ψ}\left(\frac{t-i}{s}\right)dt\;$$

By sticking out the specific wavelet ψ (·) onto the designated time series, we certainly attain $w_{s}\left(i,s\right)$. Compatibly, the key benefit of a CWT is its facility to decompose and recreate the function *x*(*t*) ∈ *L*^2^(R) as

(4) $$x\left(t\right)=\;\frac{1}{C_{\varphi }}\overset{\infty }{\underset{0}{\int }}\left[\overset{\infty }{\underset{0}{\int }}W_{x}\left(i,s\right){\text{ψ}}_{ί,s}\left(t\right)dί\right]\frac{ds}{s^{2}},s>0$$

#### 3.1.2 Wavelet transform coherence (WTC)

The squared absolute value of a wavelet cross-spectrum normalization to a single spectrum of wavelet power is well known as the WTC (Torrence and Compo, 1998). As a result, the squared wavelet coefficient is stated

(5) $$R^{2}\left(x,y\right)=\;\frac{{\left|\rho (s^{-1}W_{xy}\left(ί,s\right))\right|}^{2}}{\rho (s^{-1}{\left|W_{x}\left(i,s\right)\right|}^{2})\rho (s^{-1}{\left|W_{y}\left(ί,s\right)\right|}^{2})}$$

where ρ indicates a smoothing factor, which balances resolution and significance, and $0\leq R_{xy}^{2}\left(i,s\right)\leq 1$. A value near to 0 specifies a weak relationship, while a value near to 1 designates a strong co-movement. Wavelet analysis depicts a complete co-movement between the series in the time-frequency domain. A brighter colour indicates a stronger reliance. Because the theoretical distribution of the cross wavelet transform coefficient is unknown, the statistical implications of the relationship are investigated using the Monte Carlo procedure (Torrence and Compo, 1998).

#### 3.1.3 WTC phase difference

The wavelet transforms coherence Phase difference indicates the interruptions in the oscillation concerning the observed time series. In the case of the observed time series, the wavelet transforms coherence Phase difference shows breaks in the oscillation. Following Bloomfield et al. (2004), the phase difference between *x*(*t*) and *y*(*t*) is characterised as

(6) $$\emptyset_{xy}\left(i,s\right)=\tan^{-1}\left(\frac{J\left\{S\left(s^{-1}W_{xy}\left(i,s\right)\right)\right\}}{R\left\{S(s^{-1}W_{xy}\left(i,s\right))\right\}}\right)$$

where J and R are the imaginary operators and real operators respectively. The dimensional phase pattern in the wavelet coherence map highlights the influence of the wavelet coherence difference. Different phase patterns are distinguished using dimensional arrows.

### 3.2 Wavelet multiple correlation (WMC)

Let $X_{t}\;=\;x_{1t},\;x_{2t},\;.\;.\;.\;,x_{nt}\;$be a multivariate stochastic process and let $W_{jt}\;=\;w_{1jt},\;w_{2jt},\;.\;.\;.\;.,\;w_{njt}$ represent the resultant scale $\lambda_{j}$ wavelet coefficients attained by employing the MODWT. Fernández-Macho (2012) outlines the WMC represent by $\Omega X\left(\lambda_{j}\right)$ as a set of multiscale coherence calculated from Xt as follows. The square roots of the regression coefficient of determination (R^2^) formed by the linear combination of $w_{ijt},\;i\;=\;1,\;2,\;.\;.\;.\;,\;n$ variables for which such R^2^ is maximum is calculated at each wavelet scale $\lambda_{j}$. It is known from earlier studies that none of the auxiliary regressions ought to be run since the R^2^ conforming to the regression of a variable $z_{i}$ on a set of predictors $\left\{z_{k},\;k\;\neq \;i\right\}$ can be represented as $R_{i}^{2}\;=\;1\;–\;\rho^{-ii}$ , where $\rho^{ii}$ is the ith diagonal element of the inverse of the complete correlation matrix P. Hence WMC is achieved as in Eq. (7)

(7) $$\Omega X\left(\lambda j\right)\;={\left(1\;-\;\frac{1}{max\;diagP{\;}_{j}^{-1}}\right)}^{1/2}$$

where Pj is the (n x n) correlation matrix of $W_{jt}$.

With regards to the theory of regression, and the fitted values of $z_{i}$ as ${\hat{z}}_{t}$, then the WMC can be expressed as in Eq. (8)

(8) $$\Omega X\left(\lambda j\right)\;=\;\frac{Corr\left(w_{ijt},\;{\hat{w}}_{ijt}\right)Cov\left(w_{ijt},\;{\hat{w}}_{ijt}\right)\;}{{\left(Var\left(w_{ijt}\right)Var\left(\;{\hat{w}}_{ijt}\right)\right)}^{1/2}}$$

where $w_{ij}$ is selected to maximize $\Omega X\left(\lambda_{j}\right)\;and\;{\hat{w}}_{ijt}$ are the fitted values in the regression of $w_{ij}$ on the remaining wavelet coefficients at scale $\lambda_{j}$.

We may define WMCC as generated by allowing a lag τ between observed and fitted values at each scale $\lambda_{j}$

(9) $$\Omega X,\;\tau \left(\lambda_{j}\right)\;=\;Corr\left(w_{ijt},\;{\hat{w}}_{ijt+\tau }\right)\;=\;\frac{Cov\left(w_{ijt},\;\ddot{{\hat{w}}_{ijt+\tau }}\;\right)\;}{Var\left(w_{ijt}\right)Var\left({\hat{w}}_{ijt+\tau }\right)\;}$$

where for n = 2, WMC and WMCC converge with the standard wavelet correlation and cross-correlation.

To estimate WMC and WMCC let the realization of the multivariate stochastic process $X_{t}$ for t=1,2,...,T be $X\;=\;\left\{X_{1},\;X_{2},\;.\;.\;.\;,\;X_{T}\right\}.$ Relating a MODWT of order J to each of the univariate time series {$X_{1i}$, . . ., $X_{1T}$}, for i=1,2,...,n, the Jlength−T vectors of coefficients of MODWT ${\tilde{W}}_{j}=\;\left\{{\tilde{W}}_{j1},{\tilde{W}}_{j1},\;.\;.\;.\;,\;W{\tilde{W}}_{j,\;T-1}\right\},\;for\;j\;=\;0,\;1,\;.\;.\;.\;,\;J$ is obtained.

From Eq. (9), a nonlinear function of all n(n−1)/2 wavelet correlations of scale $\lambda_{j}$ and a steady estimator of wavelet correlation from the MODWT can be represented by

(10) $$\tilde{\Omega }X\left(\lambda_{j}\right)\;=\;{\left(1\;-\;\frac{1}{maxdiag\;{\tilde{P}}_{j}^{-1}}\right)}^{1/2}\;=\;\frac{Corr\left({\tilde{w}}_{ijt},\;{\hat{\tilde{w}}}_{ijt}\right)\;Cov\left({\tilde{w}}_{ijt},\;{\hat{\tilde{w}}}_{ijt}\right)}{{\left(Var\left({\tilde{w}}_{ijt}\right)Var\left({\hat{\tilde{w}}}_{ijt}\right)\right)}^{1/2}}$$

where ${\tilde{w}}_{ij}$ ∶ the regression of the same set of regressors $\left\{{\tilde{w}}_{kj},\;k\;\neq \;i\right\}$ maximizes the R^2^,$\;{\hat{\tilde{w}}}_{ij}$ denotes conforming fitted values, and $L_{j}\;=\;\left(2^{j}\;-\;1\right)\left(L\;-\;1\right)$ is the number of wavelet coefficients influenced by the boundary conditions associated with wavelet filter of length L and scale $\lambda_{j}$ but $\tilde{T}\;=\;T\;-\;L_{j}\;+\;1$ is the number of wavelet coefficients unaffected by the boundary conditions.

In the same vein, a consistent estimator of the WMCC can be computed as

(11) $$\tilde{\Omega }X,\;\tau \left(\lambda_{j}\right)\;=\;\frac{Corr\left({\tilde{w}}_{ijt},\;{\hat{\tilde{w}}}_{ijt+\;\tau }\right)\;Cov\left({\tilde{w}}_{ijt},\;{\hat{\tilde{w}}}_{ijt+\;\tau }\right)}{{\left(Var\left({\tilde{w}}_{ijt}\right)Var\left({\hat{\tilde{w}}}_{ijt+\;\tau }\right)\right)}^{1/2}}$$

In calculating the confidence interval (CI) of WMC, Fernández-Macho (2012) applies the transformation defined as arctan h(r), where arctan h(.) is the inverse hyperbolic tangent function for simplicity sake (Tweneboah, 2019). The confidence interval is built on a similar idea of the realization of X in the estimation of WMC and WMCC and hence for $\;\tilde{\Omega }$ $\text{X}\left(\lambda_{j}\right)$ in Eq. (11), the ${\tilde{z}}_{j}\;∼\;Fℵ(z_{j},\;{\left(T/2^{j}\;-\;3\right)}^{-1})$, where $z_{j}=\;arctan\;h\left(\Omega X\left(\lambda_{j}\right)\right),\;{\tilde{z}}_{j}\;=\;arctan\;h\left(\tilde{\Omega }\;X\left(\lambda_{j}\right)\right)$, and Fℵ symbolize the folded normal distribution. Thus, an estimate (1 − α) CI is represented by

(12) $$CI\left(1-\alpha \right)\left(\Omega X\left(\lambda_{j}\right)\right)\;=\;tanh\;\left[{\tilde{z}}_{j}\;-\;\frac{C_{2}}{{\left(\frac{T}{2^{j}}-3\right)}^{\frac{1}{2}}};\;{\tilde{z}}_{j}+\frac{C_{1}}{{\left(\frac{T}{2^{j}}-3\right)}^{\frac{1}{2}}}\right]$$

where the Fℵ critical values $C_{1}$, $C_{2}$ are: $\Omega \left(C_{1}\right)+\Omega \left(C_{1}\;-2z^{0}\right)\;=\;1-\alpha /2$ and $\Omega \left(C_{2}\right)+\Omega \left(C_{1}\;-2z^{0}\right)\;=\;2-\alpha /2$ with Ω(.) as the standard normal distribution function and $tanh\left(z^{0}\right)\;=\;\Omega_{X}^{0}\left(\lambda \right)$ as the value of some WMC formulated under a null hypothesis of the absence of correlation.

### 3.3 Data sources and description

The study employed daily stock prices of BRIC economies which are made up of Brazil (Brazil Ibovespa Index), Russia (Moscow Exchange Russia Index), India (NIFTY 500 Index), China (Shanghai Stock Exchange Composite Index) and BRIC index. We further consider the Chicago Board Options Exchange (CBOE) Volatility Index (VIX). The daily data span 2012/12/11–2021/05/28 yielding a total of 1733 observations after eliminating missing data. The suggested period was chosen to cover the aftermath of the Global Financial Crisis (GFC), the Eurozone crisis and the COVID-19 pandemic period. The daily data on BRIC economies was gleaned from the EquityRT database. The study was based on the returns of daily stock and global fear index given as $r_{t}=\text{ln}P_{t}-lnP_{t-1}$, where $r_{t}$ is the continuously compounded return, $P_{t}$ and $P_{t-1}$ are current and previous indexes respectively.

**Figure 1:**
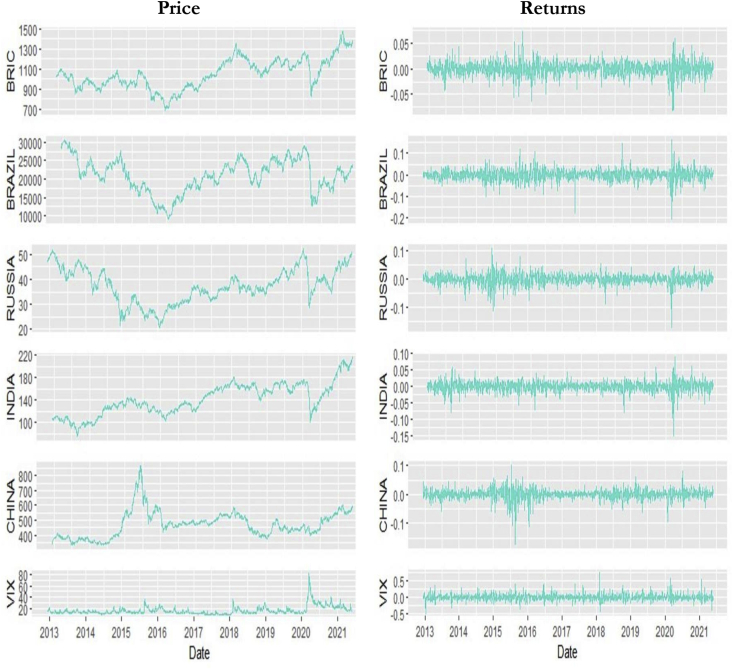
Plots of price and returns series.

Figure 1 provides the time-varying prices and log-returns of BRIC economies and VIX. It can be observed from the price series that in the early part of 2020, the price series for all markets trend upwards, after a downward spike. That is, the prices of BRIC are experiencing a rapid increase which concurs with the assertion made by Zhang et al. (2020) of markets rebound later in the COVID-19 periods since most businesses and economies have learnt how to survive. On the other hand, the price series for VIX trends downwards, after a downward spike in the latter part of 2020. This pictorially reveals the inverse relationship between stock market performance and investor fear. As a result, the variables considered in this study becomes imperative to be studied especially, during periods of uncertainty. The log-returns series in Figure 1 supports the stylised facts of asset returns thereby exhibiting volatility clustering.

Table 1 exhibits the preliminary analysis of the BRIC markets and investor fear for the study period. The skewness values reveal closer to asymmetry whilst the kurtosis values show leptokurtic behaviour in the markets and investor fear. This suggests that the data used for the study is not normally distributed. In terms of the stationarity test, the Augmented Dicky-Fuller (ADF) and the Kwiatkowski-Phillips-Schmidt-Shin (KPSS) are used. The observations from both the ADF and the KPSS reveal that all the data series explicitly fulfil the stationarity requirements. This is in line with assumptions of various autoregressive studies such as (Engle, 1982) which assumes global stationarity.

**Table 1:** Preliminary analysis.

	Mean	Std. dev	Skewness	Kurtosis	Normtest W	ADF	KPSS
**BRIC Index and Constituents**							
BRIC	0.0002	0.0126	-0.6276	6.0673	0.9296	-10.429∗∗∗	0.0860
Brazil	-0.0001	0.0241	-0.7566	10.8003	0.9022	-9.8174∗∗∗	0.0999
Russia	0.0000	0.0176	-0.9869	9.6720	0.9132	-11.1400∗∗∗	0.1614
India	0.0004	0.0146	-1.1346	11.3683	0.9023	-10.8560∗∗∗	0.0548
China	0.0003	0.0161	-1.3376	13.8307	0.8744	-11.1930∗∗∗	0.0740
**Investor expectation and fear**							
VIX	0.0000	0.0888	1.0615	7.9000	0.9063	-12.7400∗∗∗	0.0118

## 4 Results and discussion

### 4.1 Time-frequency domain

We present the bi-wavelet technique to decipher the extent of bi-causality or unidirectional relationship between two variables in time-frequency. This technique would also reveal the extent of comovements between two variables in both time and frequency domain. The horizontal axis present the time domain (calendar time) and the vertical axis present the frequency (intrinsic time or time horizons) domain. Together they form the time-frequency domain framework. The statistical interpretations and scripts for analysis were obtained from Gouhier et al. (2013). To ensure smooth interpretation of the data, right-pointing arrows and left-pointing arrows show when BRIC stock market returns and VIX are respectively in-phase (movement in the same direction) and anti-phase (movement in the opposite direction). Right-pointing arrows upwards and left-pointing arrows downwards indicate that the first variable is leading, whereas left-pointing arrows upwards and right-pointing arrows downwards indicate that the second variable is leading. The degree of interdependence between the paired series is represented by the surface colour and the colour palette. The red (warm) colour signifies parts that have major interactions, while the blue (cold) colour shows a lower series of correlations (Owusu Junior et al., 2018; Frimpong et al., 2021; Asafo-Adjei et al., 2021). The cone of influence (COI) within the biwavelet plots indicates the region where interpretation of the wavelet becomes important. The results outside the COI are insignificant since they are beyond the 95% confidence level.

**Figure 2:**
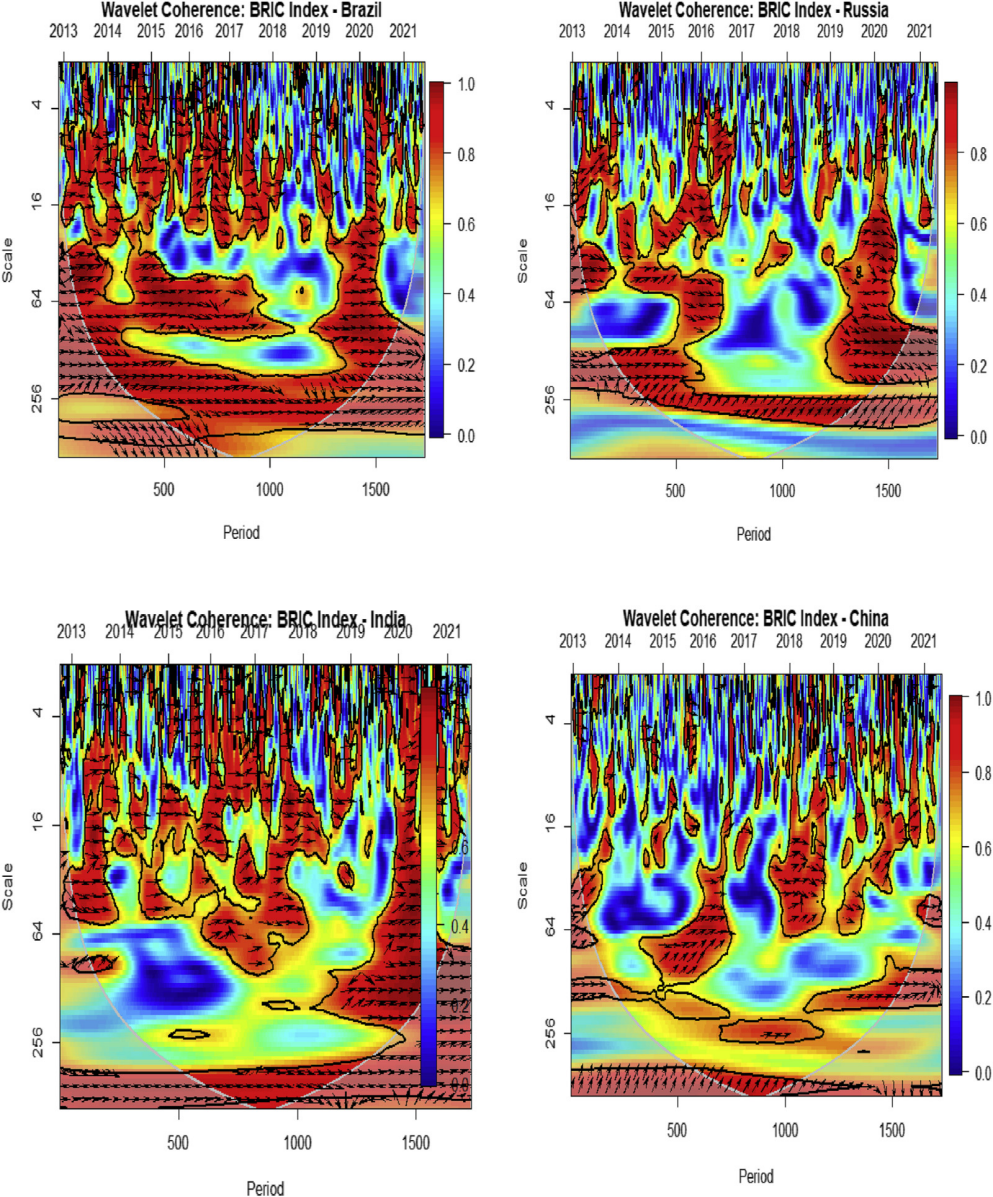
Comovements between BRIC Index and its constituents.

Figure 2 demonstrates the comovements between the BRIC index and its constituents in a time and frequency domain. The red (warm) colour in most sections of the plots signifies that there are major interactions between the BRIC index and its constituents in the short-, medium-, and long-term. The high comovements in both times and frequencies may suggest that speculative inflow has dominated financial linkages in this region. Notwithstanding, major interactions exist between the BRIC index and the stock market of Brazil. This is followed by the stock markets of India and then Russia. China has the least comovements with the BRIC index.

The right-pointing arrows indicate that comovements between the BRIC index and its constituents occur in the same direction, and are considered to be in-phase. That is, an increase or decrease in the values of BRIC stocks highly corresponds with each other. This depicts strong interdependencies among BRIC stock markets as indicated by extant literature (Dasgupta, 2014; Heliodoro et al., 2020), thereby amplifying the financial markets integration theory. Accordingly, using the BRIC index as a reliable proxy for demonstrating interdependencies with BRIC stock markets is highly confirmed. This makes portfolio diversification, safe haven and hedge benefits to be limited in the stock markets of BRIC. However, a portfolio with any one of the BRIC stock markets could be worthwhile due to the long-term integration within BRIC stocks which facilitates trade and investments.

The contribution of the BRIC stock markets to the BRIC index can also be assessed from the direction of the arrows – thus, whether upwards or downwards. Specifically, most right-pointing arrows in the comovements of the BRIC index and Brazil are downwards throughout the time-frequency domains, and considered to be unidirectional causality. That is, in the short-, medium-, and long-term, the stock market of Brazil significantly contributes to the development of the BRIC index from 2012 to 2021, and it is also the first variable to respond to shocks before the BRIC index. The stock market of Russia records a mixture of bidirectional causality with the BRIC index. Specifically, in the medium-term, the stock market of Russia drives the BRIC index, but otherwise for the short-, and long-term, except for 2020. In 2020, the stock market of Russia drives the BRIC index, and may be attributed to the COVID-19 pandemic which has distorted most economic and financial activities. Right-pointing arrows in the case of India and China are mostly upwards than downwards. Thus, the BRIC index rather leads most of the comovements with the stock markets of India and China. In this regard, the BRIC index would be the first variable to respond to shocks, ex-ante the stock markets of India and China.

**Figure 3:**
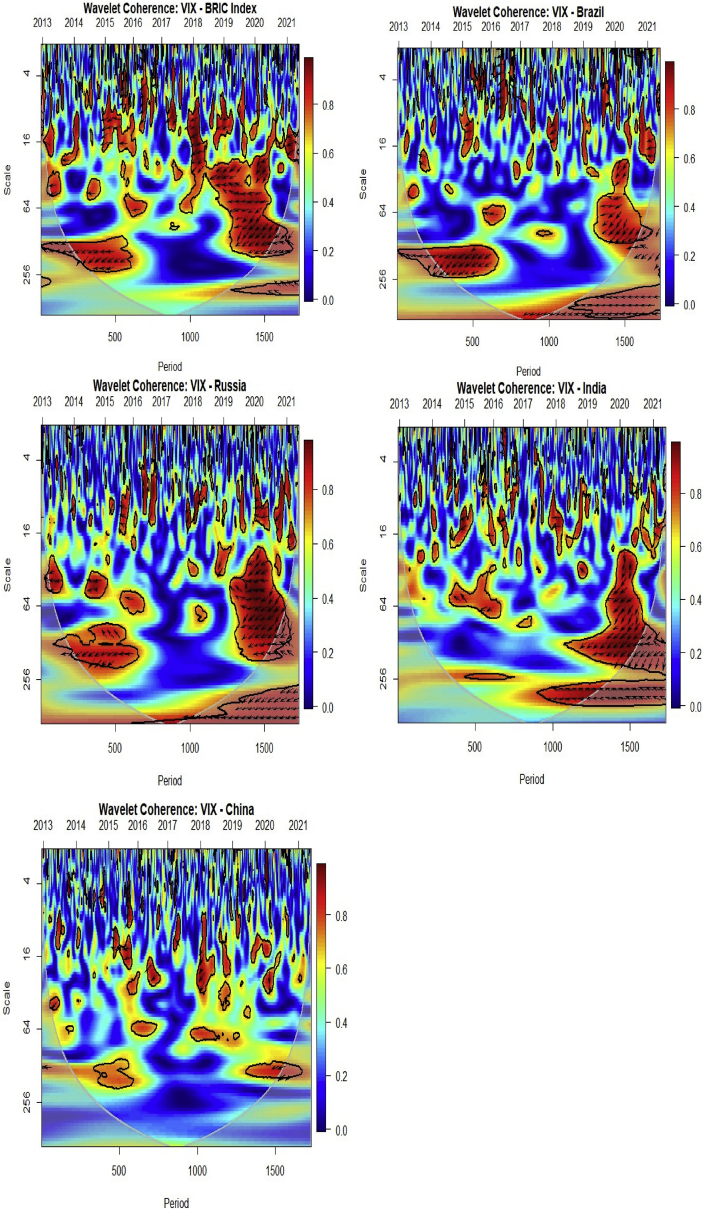
Comovements between investor fear and BRIC.

Figure 3 exhibits the comovements between the US VIX and the stock markets of BRIC through the bi-wavelet technique. Analysis from Figure 3 differs from Figure 2 by employing the US VIX to assess its comovements or degree of causality with the BRIC stocks in time-frequency domain.

The red (warm) colour in the plots signifies that there are major interactions between the US VIX and the stock markets of BRIC in the short-, medium-, and long-term. Notwithstanding, major interactions exist between the US VIX and the BRIC index. This is followed by the stock markets of Russia and then Brazil. China has the least comovements with the US VIX. This is not surprising because, the stock markets of China exhibits less comovements with the stock markets of US (Aloui et al., 2011; Morales and Gassie-Falzone, 2014a, Morales and Gassie-Falzone, 2014b; Kannadhasan and Das, 2019), irrespective of market conditions. This may be attributable to the US-Chinese Trade war which has resulted to their limited interconnectedness. As such, markets in China provide rewarding grounds for short-term and long-term investors. To global investors, a portfolio with Chinese stocks and any one of the remaining stock markets of BRIC offers safe haven, diversification and hedge benefits depending on the market conditions (see, Baur and Lucey, 2010).

The left-pointing arrows indicate that comovements between the US VIX and BRIC occur in the reverse direction, and are considered to be anti-phase. That is, an increase or decrease in the values of BRIC stocks highly alternates with the VIX. This depicts the adverse interdependencies between the US VIX and BRIC stock markets as indicated by extant literature (Schwert, 1989; Engle and Ng, 1993; Zakoian, 1994; Bekaert and Wu, 2000, Sarwar, 2012, Bhuyan et al., 2016, etc.), thereby amplifying the financial instability hypothesis of Minsky (1975, 1986, 1992) and the asymmetric volatility phenomenon. Accordingly, the US volatility index is an effective proxy for gauging fear into BRIC markets.

The study also reveals that the VIX drives the comovements with BRIC stocks. Specifically, most left-pointing arrows are downwards throughout the time-frequency domains, and considered to be unidirectional causality. That is, in the short-, medium-, and long-term, the stock market of the US VIX significantly drives the drawdowns of BRIC stocks, and the former is also the first variable to respond to shocks from each market. Beyond 2019, the strong adverse comovements between the VIX and stock markets of BRIC may be attributed to the COVID-19 pandemic which has heightened the asymmetric volatility phenomenon. This makes the stock markets of BRIC more preferrerd to receiving safe haven benefits from the VIX futures (Shahzad et al., 2021) in times of market stress.

### 4.2 Frequency-domain

**Figure 4:**
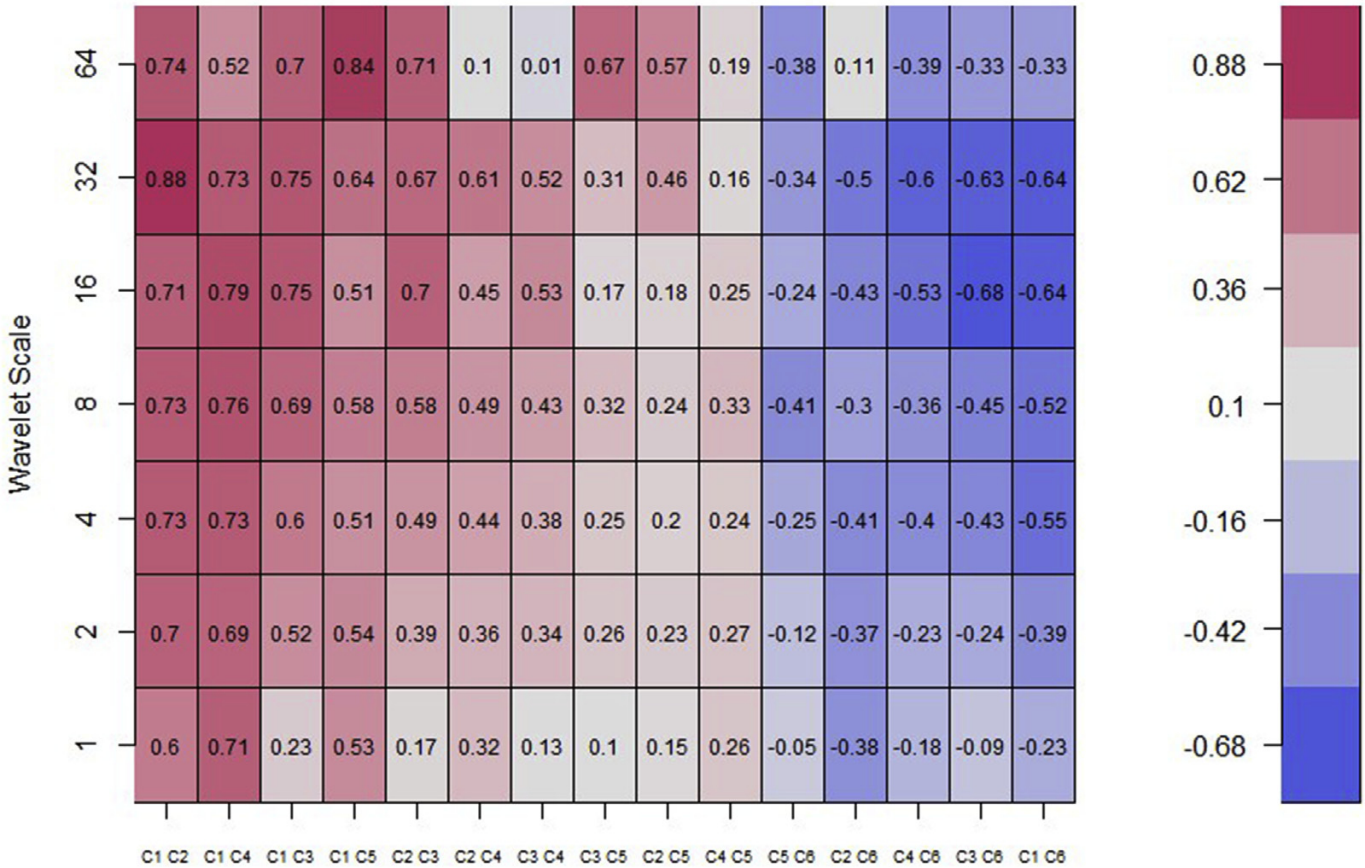
Wavelet bivariate correlations matrix (2012/12/11–2021/05/28). *The codes for the variables are BRIC index (C1), Brazil (C2), Russia (C3), India (C4), China (C5) and VIX (C6)*.

**Figure 5:**
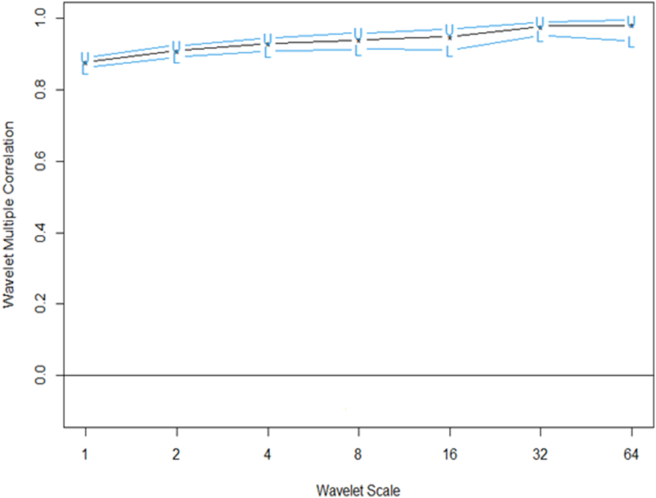
Wavelet multiple correlation among BRIC index, BRIC constituents stocks and VIX returns (2012/12/11–2021/05/28). *U-upper limits, L-lower (at 95% confidence interval)*.

**Figure 6:**
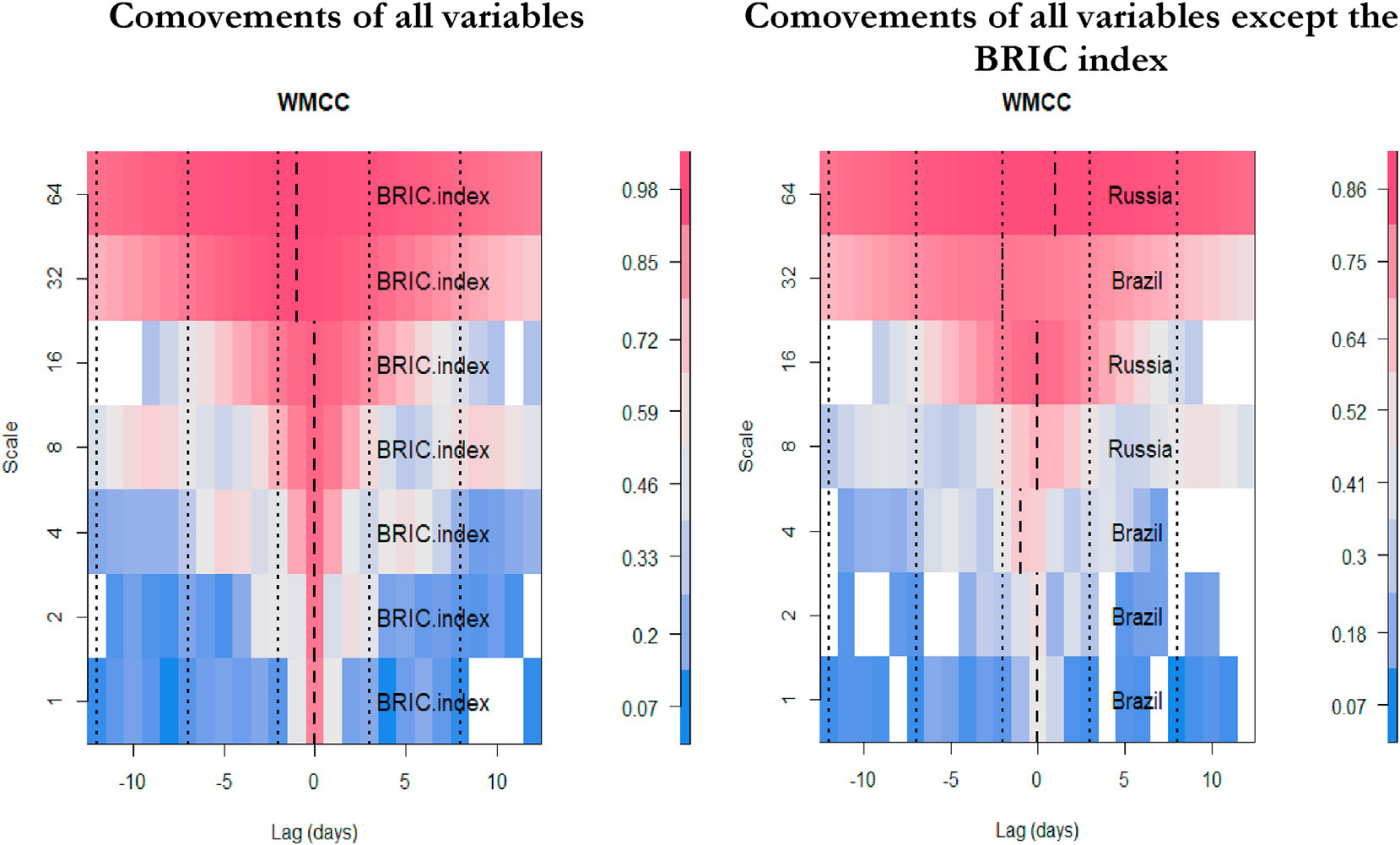
Wavelet multiple cross-correlation between among BRIC index, BRIC constituents stocks and VIX returns (2012/12/11–2021/05/28).

We present the multiple wavelet analysis for BRIC stock markets and investor expectations and fear (VIX). The meaning of the scales in the care of data frequency of 5 days per week, lj,j=1...7, of the wavelet factors are connected to times of, respectively, “2–4 days (intraweek scales), 4–8 days (weekly scale), 8–16 days (fortnightly scale), 16–32 days (monthly scale), 32–64 days (monthly to quarterly scale), 64–128 days (quarterly to biannual scale), and 128–256 days (biannual to annual scale)” for scales 1–64 respectively for Figures 4, 5, and 6 (Tweneboah et al., 2019; Asafo-Adjei et al., 2021).

#### 4.2.1 Wavelet bivariate correlations matrix

At 5 wavelet scales, the bivariate contemporary correlations are considered. The codes for the variables are the BRIC index (C1), Brazil (C2), Russia (C3), India (C4), China (C5) and the US VIX (C6). For calculating wavelet correlation coefficients, the horizontal axis displays the possible combinations. If we switch from left to right, the similarities between the pairs of BRIC index, Brazil, Russia, India, China and the US VIX nexus become weaker. On the vertical axis, the wavelet scales reflect frequencies. Thus, the bivariate contemporary correlation matrix is frequency-dependent other than having the features of both time-frequency domains (Asafo-Adjei et al., 2021).

From Figure 4, we present the wavelet correlation matrix for BRIC index, Brazil, Russia, India, China and the US VIX returns across the seven wavelet scales, which does not seem to differ significantly from the bivariate analysis. We find a mix of positive and negative relationships among the pairs. The BRIC index and stock market of Brazil demonstrated the maximum degrees of co-movement with coefficients fluctuating over 0.6 to 0.88 at diverse time scales averaging 0.73 indicating no extreme correlational values. Thus, the comovements between the BRIC index and Brazil is high and symmetrical across all the wavelet scales. This is followed by the comovements between BRIC index and India, BRIC index and Russia, and BRIC index and China. In this regard, the constituents of BRIC and their index depict significant comovements throughout the wavelet scales. Also, between the constituents, there exist positive comovements rendering portfolio diversification, safe haven and hedge benefits to be impracticable within this region. Notwithstanding, an investor could easily select any one of the stock markets within this region to allocate their investments since they depict similar comovements.

However, the dynamics of the comovements changed when the US VIX as a measure of investor expectation and fear was considered. Thus, the US VIX is capable of gauging fear into BRIC markets in the short-, medium-, and long-term. Throughout the wavelet scales, the VIX demonstrates very weak negative comovements with– BRIC index, Russia, India, Brazil and China. Thus, the US VIX could act as a hedging instrument for BRIC stocks (Shahzad et al., 2021).

### 4.3 Wavelet multiple correlations (WMC)

Figure 5 (Table 2) and Figure 6 (Table 3) denote the wavelet multiple correlations and wavelet multiple cross-correlations respectively for the BRIC index, constituents of BRIC and the US VIX nexus returns series into frequency localization by the MODWT (Fernández-Macho, 2012).

**Table 2:** Wavelet multiple correlations (WMC).

Scale	WMC “lower”	Correlation	WMC “upper”
1	0.861078322	0.877367869	0.89185834
2	0.891278969	0.909154808	0.924209296
3	0.908644049	0.929402708	0.945578905
4	0.913373872	0.940085438	0.958737993
5	0.911657853	0.948004749	0.969634861
6	0.951752694	0.978032525	0.990070814
7	0.936052964	**0.981056815**	0.994478738

Bold value indicates that all values are significant at 1%.

**Table 3:** Wavelet multiple cross correlations (WMCC).

Comovements of all Variables			
Scale	Localizations	Time Lag (days)	Leading/Lagging variable
1	0.877367869	0	BRIC
2	0.909154808	0	BRIC
3	0.929402708	0	BRIC
4	0.940085438	0	BRIC
5	0.948004749	0	BRIC
6	0.979979043	-1	BRIC
7	**0.981980385**	-1	BRIC
**Comovements except BRIC**			
1	0.476355033	0	Brazil
2	0.527423426	0	Brazil
3	0.600174603	-1	Brazil
4	0.662317654	0	Russia
5	0.822349479	0	Russia
6	0.795911920	-2	Brazil
7	**0.863568320**	1	Russia

Bold value indicates that all values are significant at 1%.

Figure 5 and Table 2 establish the degree of integration between the study variables from the short-term to the long-term dynamics. It does not depict the leading or lagging variable, but indicates the general comovements among the study variables. The degree of integration is comparatively high for the daily return series reaching as high as approximately 98% for the wavelet multiple correlations, 94% for the lower panel and 99% for the upper panel. This suggests that many correlations are increasing continuously over time. Thus, daily returns of one variable can be explained by the remaining five (5) variables to a degree of about 98% from daily, leading up to scale 64 interdependence. In other words, there exist high interdependencies irrespective of the presence of the US VIX which gauges adverse shocks in the BRIC stock markets. This outcome corroborates the study of Kannadhasan and Das (2019) who found strong comovements in BRICS economies. They indicated that the strong comovements entail a state near to perfect integration, and may limit the benefits of arbitrage and portfolio diversification. This implies that, when there is a high integration between stock markets, the adverse pervasive nature of volatility is unlikely to penetrate, except for each stock in isolation as shown from Figure 3. We strongly agree the assertion made by prior literature that deepening market integration has increased the vulnerability of markets to external shocks, since asset prices will be explained by common factors (Bekaert and Harvey, 1995; Baele, 2005; Dewandaru et al., 2014). However, we advocate that, the distinctive and economic financial systems, capacities and capabilities of BRIC markets may minimise the adverse impact of the volatilities.

### 4.4 Wavelet multiple cross correlations (WMCC)

The wavelet multiple cross-correlation coefficients are presented in Table 3 depicting seven wavelet scales. From Figure 6, the scales at the y-axis have similar meanings as indicated at the preliminary stage of the multiple wavelet analysis discussion. The x-axis, however, represents the lag length of the series. In this case, 10 days for positive and negative lags each. We need both positive and negative shocks to confirm the potential leading and lagging variables. Localisations at positive lag denote lagging variable and negative lag denote leading variable at the respective scales. At the zero-lag of localisation (dashed) lines, there is no lead or lag. Localisation implies the maximum values in the linear combination of all variables at the wavelet scales, which are indicated by dashed lines within the dotted lines (at all lags). A variable listed on a scale indicates the variable with the potential to lead or lag all the other variables. It implies that, at that scale, it has the maximum value in the linear combination of all the variables at the respective scales. When a dashed line accompanies a listed variable in the heatmap, then it becomes an actual lead (negative lag) or lag (positive lag) unless the dashed line is on the zero-lag which implies neither lead nor lag. Accordingly, the economic implication of the wavelet multiple cross-correlations (WMCC) is that it indicates the degree of interdependence between the variables, and determines the most influential variable at a specified wavelet scale to act as either a leading (first mover to respond to shocks) or lagging (the last variable to respond to shocks after the remaining variables) variable.

The comovements among all the variables reveal that the BRIC index has the potential to lead or lag from scales 1 to 16 representing intraweek to quarterly scales respectively (at lag 0). This is within the short-, and medium-term. However, in the long-term represented by scales 32 and 64, indicating quarterly to annual scales respectively, the BRIC index leads the remaining variables (at lag -1). It goes to suggest that the BRIC index is a good indicator to represent its regional bloc. In terms of stock market performance, the BRIC index would be the first variable to respond to shocks before the constituents. Thus, the composite of the few shocks within the constituents has a significant impact on the BRIC index to be the first variable to respond to shocks. In other words, in times of uncertainty, the BRIC index would fall faster than its constituents and rise faster during a boom.

In a similar fashion, the comovements between the other variables except the BRIC index depicts that the stock markets of Brazil and Russia have the potential to lead or lag the remaining other variables for most scales. Specifically, the stock market of Brazil has the potential to lead or lag for intraweek and weekly scales, but leads at fortnightly and quarterly to biannual scales. Thus, aside the BRIC index, the stock market of Brazil is the next variable to respond to shocks, and would indicate high connectedness with the BRIC index as indicated by the bi-wavelet technique. The stock market of Russia has the potential to lead or lag at monthly to quarterly scales, but lags at the annual scale. Thus, the stock market of Russia would be the last variable to respond to shocks in the long term.

## 5 Conclusion

We present four national stock markets to assess their comovements with their corresponding index variable within the BRIC economies. Also, we include the US VIX to gauge investor expectations and fear in the discussion of BRIC markets comovements in a time-frequency domain. Moreover, we examined the degree of integration or interdependencies among the stock markets of BRIC constituents, BRIC index, and the US VIX simultaneously to provide a full picture of the nexus. Hence, the wavelet techniques – the bi-wavelet and wavelet multiple were employed to further respond to the heterogeneous nature of market participants and their adaptive behaviours (Boateng et al., 2021). As indicated by Tan et al. (2019), time scale analysis in emerging markets cannot be ignored due to their increasing level of trade and investments. In this regard, the current study is among the very few empirical studies that assess the comovements between and/or among the stock markets of BRIC constituents, BRIC index, and the US VIX via the wavelet techniques, and inclusive of the COVID-19 pandemic.

The bi-wavelet technique revealed that there are high interdependencies between the BRIC index and its constituents which was concentrated in the short-, medium-, and long-term. In addition, comovements between the BRIC index and its constituents were positive and significant throughout the wavelet timescales. Thus, in times of uncertainties, drawdowns in the performance of the constituents are, however, captured in the BRIC index. Notwithstanding, we found the BRIC index to be the first variable to respond to shocks when all the study variables were considered in the Wavelet multiple cross-correlations. In a similar fashion, when the BRIC index was eliminated from the wavelet multiple cross-correlations, the stock market of Brazil was the next to respond to shocks. On the other hand, Russia's stock market is lagging in the long run.

Further, we found investor expectation and fear to have an adverse relationship with BRIC economies in the medium-, and long-term, especially, beyond 2019. This indicates that in times of uncertainty the comovements between the US VIX and the stock markets of BRIC depict large drawdowns than upward movements to support the stylised facts of asset returns (Adam and Owusu Junior, 2017). This may be attributed to the COVID-19 pandemic which has distorted global financial and economic activities. It was also not surprising to see the US VIX driving the individual stock markets at most wavelet time scales. Thus, the causality between the US VIX and BRIC stocks was found to be unidirectional. As a result, BRIC returns are typically connected with higher (downward) adjustments of the US volatility index. Contrarily, the US VIX could not drive the stock markets of BRIC when the wavelet multiple analysis was considered. This is not overwhelming because, when there are strong interdependencies between BRIC markets, participants may enjoy their long-term relationships.

Findings from the study imply that global investors can select any of the stock markets in BRIC to allocate their investments due to their strong interdependencies which facilitate trade and investments. However, portfolio diversification, safe haven and hedge benefits within this region may be minimal due to their high integration with the BRIC index which demonstrates positive significant comovements. In that case, any adverse movement in the BRIC index is reflected in its constituents, because it was considered to be the first variable to respond to shocks. It is recommended that investors hedge against volatilities in the BRIC stock markets using the Chicago Board Options Exchange volatility index due to the existence of asymmetric volatility dynamics within the study findings.

Owing to the significant comovements within the study variables, further studies could assess the flow of information between them. This may be executed at diverse investment scales and tail events since multi-scales at lower probabilities could have richer information in financial time series (Asafo-Adjei et al., 2021). As a result, any appropriate decomposition technique in addition to the Rényi effective transfer entropy could be useful.

## Declarations

### Author contribution statement

All authors listed have significantly contributed to the development and the writing of this article.

### Funding statement

This research did not receive any specific grant from funding agencies in the public, commercial, or not-for-profit sectors.

### Data availability statement

Data included in article/supplementary material/referenced in article.

### Declaration of interests statement

The authors declare no conflict of interest.

### Additional information

No additional information is available for this paper.
